# Synthesis of Pt–MoS_2_ with enhanced photothermal and peroxidase-like properties and its antibacterial application†

**DOI:** 10.1039/d4ra05487c

**Published:** 2024-09-18

**Authors:** Liangyu Li, Yueqin Zhang, Yumeng Liu, Yaojuan Wu, Xiao Wang, Lidong Cao, Xia Feng

**Affiliations:** a Department of Nursing, Zhejiang Provincial People's Hospital, Affiliated People's Hospital, Hangzhou Medical College Hangzhou China; b Department of Hepatobiliary & Pancreatic Surgery and Minimally Invasive Surgery, Zhejiang Provincial People's Hospital, Affiliated People's Hospital, Hangzhou Medical College Hangzhou China; c College of Mechanical Engineering, Zhejiang University Hangzhou China; d School of Public Health, Hangzhou Medical College Hangzhou China

## Abstract

Despite tremendous efforts, bacterial infection and contamination remain a major clinical challenge to modern humans. Nanozyme materials with stimuli-responsive properties are expected to be powerful tools for the next generation of antibacterial therapy. Here, MoS_2_ nanosheet was firstly prepared by liquid phase exfoliation method, and Pt–MoS_2_ hybrid biomaterial was then successfully synthesized by a simple self-reduction method. The Pt decoration significantly improves the photothermal effect of MoS_2_ nanosheet under 808 nm NIR laser irradiation. Besides, benefiting from the formation of heterogeneous structure, the Pt–MoS_2_ has significantly enhanced peroxidase mimetic catalytic activity, which can kill bacteria through catalysis of H_2_O_2_ to generate antimicrobial hydroxyl radicals. Moreover, the temperature rise brought about by NIR laser stimulation further amplifies the nanozyme activity of the composites. After treatment by the synergistic platform, both *Staphylococcus aureus* and *Escherichia coli* can be effectively inhibited, demonstrating its broad-spectrum antibacterial properties. In addition, the developed antibacterial Pt–MoS_2_ nanozyme have the excellent biocompatibility, which makes them well suited for infection elimination in biological systems. Overall, this work shows great potential for rationally combining the multiple functions of MoS_2_-based nanomaterials for synergistic antibacterial therapy. In the future, the Pt–MoS_2_ nanozyme may find wider applications in areas such as personal healthcare or surface disinfection treatment of medical devices.

## Abstract 2

Despite tremendous efforts, bacterial infection and contamination remain a major clinical challenge to modern humans.

## Graphical abstract

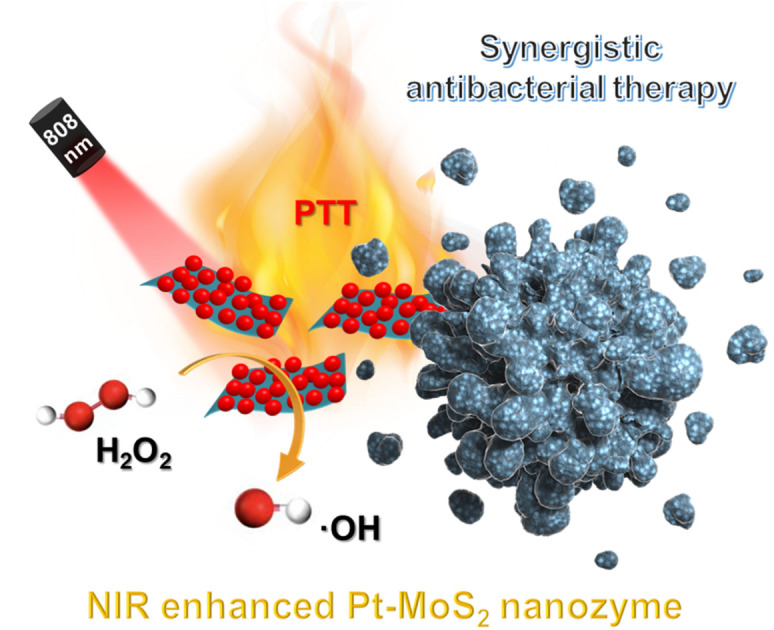

## 1. Introduction

Consistently, the risk of bacterial infection or contamination has become one of the most serious health problems in the world.^1,2^ Bacterial infections cause millions of people to get sick and die every year, and the long-term overuse of antibiotics leads to the emergence of drug-resistant microorganisms and even super bacteria, which brings huge environmental pollution and economic burden.^3^ In order to solve this problem, people have made a lot of efforts to develop antibiotic alternatives against bacterial infections.^4^ Recently, the rapid development of nanotechnology provides a promising strategy for the fight against drug-resistant bacteria.^4,5^ Compared with traditional antibiotics, nanomaterials allow rationally engineering design, such as element composition, size control, surface modification and stimulus response characteristics, for constructing sustained and broad-spectrum antibacterial platform without development of drug resistance.^6^ In recent years, some novel nanomaterial-mediated platforms such as photothermal therapy (PTT), chemodynamic therapy (CDT), and photodynamic therapy (PDT) are emerging for effective antibacterial treatment.^7–9^

In biomedical fields, enzyme-catalyzed reactions have a wide range of applications such as sensing, imaging and therapy.^10^ Recent studies have confirmed that natural enzyme-catalyzed systems in organisms can be used to inhibit bacterial growth.^11^ For example, natural peroxidases (POD) and oxidases (OXD) can catalyze their substrates for production of a variety of reactive oxygen species (ROS) to act as bactericides.^12^ However, these natural enzymes have problems of expensive price, complicated preparation and purification procedures, low stability and low recycling rate, which limit the breadth of their applications.^13^ Nanozymes, defined as nanomaterials capable of mimicking the biocatalytic activity of natural enzymes, have received increasing attention in recent years owing to the advantages of simple preparation, high stability, easy purification, low price and good recyclability.^14–16^ To date, many nanomaterials, including metals,^17,18^ metal oxides/sulfides,^19^ carbon-based materials,^20^ and metal–organic frameworks (MOFs),^21^ have been demonstrated to own catalytic ability and act as one or more natural enzymes. However, the catalytic efficiency of nanozymes in complex biological microenvironments is still insufficient and needs to be further improved to play catalytic roles. In particular, the design of multifunctional nanozymes in combination with other non-invasive therapeutics are also of great value for the development of smart integrated antibacterial therapy.^22^

Among various nanomaterials, MoS_2_ has been widely explored for antibacterial treatment due to its low cost, green synthesis, high chemical stability, high catalytic activity, good biocompatibility and good near-infrared (NIR) photothermal conversion property.^23,24^ In addition, the modification of MoS_2_ can further improve and expand its application fields. Typically, hybridization of MoS_2_ with other functional material can also greatly improve its antibacterial properties. For example, Wang *et al.* designed and synthesized Ag–MoS_2_ nanomaterials, which have POD-like enzyme activity and can decompose hydrogen peroxide to produce antibacterial hydroxyl radicals (˙OH).^25^ Moreover, with the help of Ag^+^, the antibacterial effect of Ag–MoS_2_ nanomaterials was further enhanced and synergistic antibacterial therapy was achieved. In addition, other nanomaterials including Au, TiO_2_ and MOF are also used to be composited with MoS_2_ for significant enhancement of antibacterial properties.^26–28^ As an important class of noble metal, Pt nanomaterials have many applications in nanozyme catalysis and photothermal therapy due to their ultra-fine structure, simple preparation, high stability, biocompatibility and excellent physical and chemical properties.^29,30^ The incorporation of Pt with MoS_2_ nanosheet is expected to improve the photothermal and enzyme-like catalytic activities of MoS_2_ alone, further improving the antibacterial performance from multiple aspects. However, as we know, there is currently no investigation on the preparation and application of NIR-triggered Pt–MoS_2_ nanozyme for antibacterial treatment.

Based on the above considerations, in the present work, we developed a unique Pt–MoS_2_ nanozyme with NIR-enhanced properties for synergistic antibacterial application. Firstly, morphologically uniform MoS_2_ nanoflakes were obtained by the classical liquid phase exfoliation (LPE) method. Following this, using polyvinylpyrrolidone (PVP) as a stabilizer and H_2_PtCl_6_ as a metal source, we have successfully constructed the Pt–MoS_2_ composite by *in situ* growing Pt nanomaterials on MoS_2_ nanoflakes *via* a facile and green method with the assistance of the spontaneous reductive properties of MoS_2_. The prepared Pt–MoS_2_ composite material not only have the advantage of simple synthesis, but also have enhanced NIR absorption and photothermal conversion ability with POD-like enzyme catalytic properties than MoS_2_ alone. The POD-like catalytic activity of the Pt–MoS_2_ was further improved under the stimulation of NIR laser at 808 nm, so that they could catalyze the formation of highly toxic ˙OH from H_2_O_2_ more effectively. The *in vitro* antimicrobial experiments further confirmed the efficient bactericidal ability of the combination therapy, with an inhibition rate of over 95% for both *Staphylococcus aureus* (*S.aureus*) and *Escherichia coli* (*E.coli*) what's more, the novel Pt–MoS_2_ platform showed good biological compatibility in cellular experiments. Overall, we believe that the NIR photothermally enhanced Pt–MoS_2_ nanomaterials developed in this work can be used as effective stimuli-responsive tools for safe and efficient antibacteria and disinfection in the post-antibiotic era, which may find more potential applications in wound infection and clinical care.

## 2. Experimental methods

### 2.1. Chemicals and materials

Ultrapure water (18.2 MΩ cm) was applied through the experiments to prepare all the solutions. Phosphate buffered saline (PBS), 3,3′,5,5′-tetramethylbenzidine (TMB) and molybdenum disulfide (MoS_2_) was obtained from Macklin (China). Chloroplatinic acid hexahydrate (H_2_PtCl_6_·6H_2_O), acetic acid (HAC), H_2_O_2_ (30 wt%), hydrochloric acid (HCl), sodium hydroxide (NaOH), and sodium acetate (NaAC) were purchased from Sinopharm Chemical Reagent Co., Ltd, Gram-positive *S. aureus* (ATCC 29213) and Gram-negative *E. coli* (ATCC 25922) was obtained from the American Type Culture Collection (USA). The live/dead bacterial viability kit was obtained from Beyotime (China). The EDTA-stabilized human blood (1 mL) was collected from Zhejiang Provincial People's Hospital and informed consent was obtained from the volunteers. The above experiments were conducted in accordance with relevant laws and guidelines of the hospital, and were approved by the Ethics Committee of Zhejiang Provincial People's Hospital (approval number QT2023310).

### 2.2. Preparation of Pt–MoS_2_

First, the MoS_2_ nanoflake was prepared by typical LPE method with slight modification.^31,32^ Briefly, 0.3 g of MoS_2_ powder was firstly added into 30 mL 45% ethanol aqueous solution. Then, the above solution was sonicated at 500 w for 4 h in an ice-water bath. After that, MoS_2_ nanoflake was obtained and then centrifuged at 4500 rpm for 30 min and the supernatant was collected in water for later use. To prepare Pt–MoS_2_, the as-prepared MoS_2_ (2 mL) was mixed with PVP (0.01 g) for stirring 10 min at 60 °C. Next, 50 µL of H_2_PtCl_6_ (50 mM) was added to the above mixture for reacting another 2 hours. After that, the dark brown Pt–MoS_2_ was collected and washed three times with water by centrifugation, and the excess reactants and PVP were also removed at the same time. Finally, the Pt–MoS_2_ was placed at 4 °C for further use.

### 2.3. Characterization apparatus

Transmission electron microscopy (TEM) images was carried out on a H-7650 (Hitachi). UV-vis spectra were acquired by a UV-2450 spectrometer (Shimadzu). Raman spectra were performed by a Microscopic Raman spectrometer (Renishaw). The zeta potential and diameter were tested on a laser particle size meter (Zetasizer Nano-ZS90). X-ray photoelectron spectroscopy (XPS) measurements were performed on ESCALAB 250XI (Thermo Fisher). The temperature and thermal images were obtained and analyzed by FLIR ONE thermal imager (FLIR Systems, Inc, Wilsonville, OR).

### 2.4. Photothermal activity test

To evaluate the NIR photothermal performance, 200 µg mL^−1^ of MoS_2_ and Pt–MoS_2_ in a 48-well plate was used to investigate and the photothermal activity under 808 nm NIR light. As shown in the Fig. S1,† the 808 nm laser is illuminated vertically in a 48-well plate pre-placed with the nanomaterial. Because the laser output was filtered and collimated, the diameter of light spot is 10 mm at irradiation distance of 10 cm. The corresponding power density can be obtained by adjusting the laser power. The influence of Pt–MoS_2_ concentration was further evaluated by recording the temperature of aqueous solution under the 808 nm NIR light. To characterize the reversible photothermal ability, 0.8 mL Pt–MoS_2_ (200 µg mL^−1^) and 1 W cm^−2^ of 808 nm laser power were selected to perform three heating–cooling cycles.

### 2.5. Nanozyme activity test

To test the POD-like catalytic activity, 5 mM TMB was first mixed with 10 mM H_2_O_2_ in NaAC–HAc buffer at pH 3.0. After that, the MoS_2_ and Pt–MoS_2_ (100 µL, 1 mg mL^−1^) were added and reacted at room temperature for 10 min. The UV-vis absorbance spectra and the time-dependent absorbance value was recorded by UV spectrometer. To study the influence of pH and temperature for the POD-like activity, Pt–MoS_2_ (10 µL, 1 mg mL^−1^) was added to a centrifuge tube containing HAc–NaAc buffer solution (150 µL) with different pH (2–8) and temperature (20–60 °C). The catalytic kinetics of Pt–MoS_2_ at 652 nm was tested based on the absorbance value change of system. The kinetics parameter including Michaelis–Menten constant (*K*_m_) and maximum reaction rate (*V*_max_) were obtained according to the double reciprocal curve of the Michaelis–Menten equation:
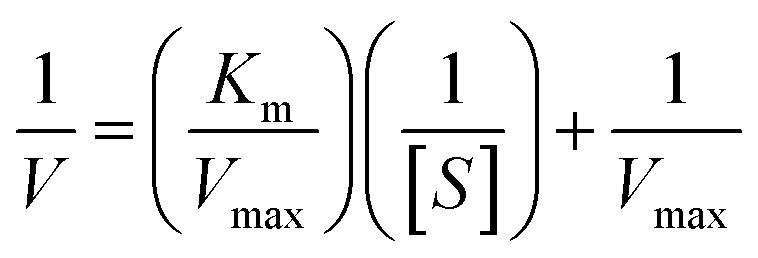
where *V* is the initial reaction speed, *V*_max_ is the maximal velocity, [*S*] is the H_2_O_2_ concentration, and *K*_m_ is the Michaelis constant.

### 2.6. Bacterial culture and antibacterial activity tests

In this study, Luria-Bertani broth medium (LB) was used to culture *S. aureus* and *E. coli*. First, the bacteria were cultured overnight in LB medium at 37 °C. When the optical density of bacteria reaches the range of 0.6–0.8, the culture is stopped. Next, the bacteria were collected by centrifugation and diluted with PBS. Hydrogen peroxide (2 mM) or nanomaterials (200 µg mL^−1^) were mixed with *S. aureus* or *E. coli* with a bacterial density of 10^6^ colony-forming units (CFU mL^−1^) and incubated at 37 °C for 3 hours For Pt–MoS_2_ group, 808 nm NIR laser (1.0 W cm^−2^) was utilized to heat the antibacterial system in LB medium. Then, the treated bacteria were washed with PBS and inoculated on agar plate overnight. The measurement of OD_600_ by a microplate reader was taken to indicate bacterial viability.

### 2.7. Morphology observation and live/dead fluorescent staining

To observe the bacterial morphologies, *S. aureus* or *E. coli* were first washed with PBS, and then glutaraldehyde was used to fix them up. After that, the *S. aureus* or *E. coli* were dehydrated subsequently by 35%, 60%, 75%, 85%, 95%, 100% ethanol solutions. Finally, the scanning electron microscope (SEM) images of these cells were obtained to using an SU3500 SEM (Hitachi, Japan). For fluorescent Staining, after treated with several groups, *S. aureus* was stained with SYTO 9/PI dye in the dark for 20 min at 25 °C. Then, the fluorescent images of stained bacterial cells were recorded using a confocal laser microscope (Zeiss).

### 2.8. Cytocompatibility test

The cytocompatibility of Pt–MoS_2_ was determined by 3-(4,5)-dimethylthiahiazo (-z-y1)-3,5-di-phenytetrazoliumromide (MTT) assay. Firstly, the NIH3T3 cells were incubated in the 96-well plate for 24 h. Then, high glucose medium was added and incubated with Pt–MoS_2_ at different concentrations for another 24 h or 48 h. Following this, culture medium was replaced with MTT and incubated for another 4 h. During the assay, MTT can be reduced by dehydrogenases within the mitochondria from living cells to form the dark purple product formazan. After that, dimethyl sulfoxide (DMSO) was mixed for 15 min and the optical density was recorded at 570 nm, maximum absorbance of dark purple product formazan using the microplate reader. Three control groups were set at the same time. The cell viability was calculated as follows: Cell viability (%) = OD_Pt–MoS_2__/OD_PBS_ × 100%, where OD_Pt–MoS_2__ and OD_control_ represented the optical density of cells treated with the Pt–MoS_2_ and PBS, respectively.

### 2.9. Hemolysis assay

Firstly, red blood cells (RBCs) was separated from blood sample by centrifugation at 1500 rpm for 15 min. Subsequently, RBCs were washed three times and diluted by PBS when the OD value was 0.5 ± 0.05. After that, RBCs were incubated with Pt–MoS_2_ at different concentrations at 37 °C for 2 h. The mixture was then centrifuged at 1500 rpm for 15 min. and the supernatant's optical density was recorded at 570 nm using the microplate reader. The hemolysis ratio was calculated based on previous work.^33^

## 3. Results and discussion

### 3.1. Synthesis and characterization of Pt–MoS_2_

**Fig. 1:**
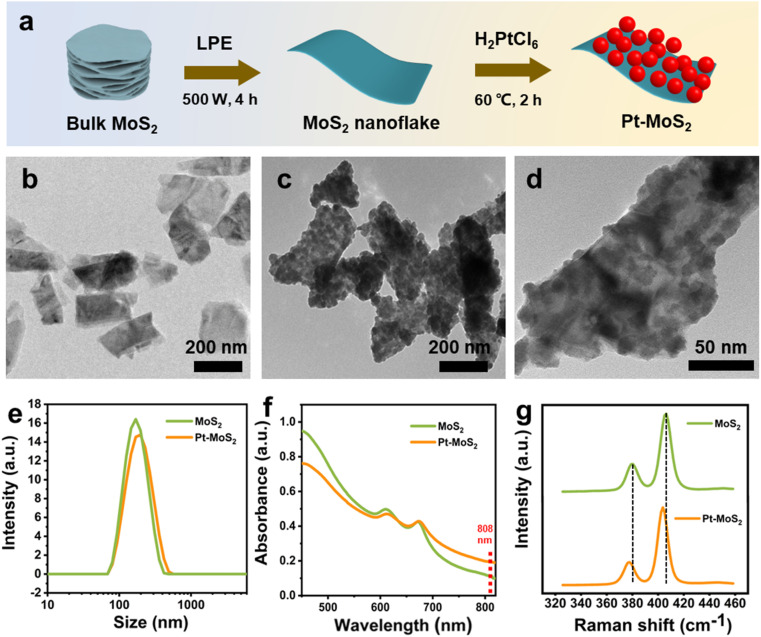
(a) Schematic illustration for the preparation process of the MoS_2_ nanoflake and Pt–MoS_2_ nanocomposite. TEM images of MoS_2_ nanoflake (b) and Pt–MoS_2_ nanocomposite (c and d). (e) DLS analysis for hydrodynamic sizes of MoS_2_ and Pt–MoS_2_. (f) UV-vis absorbance spectra of MoS_2_ and Pt–MoS_2_ at 100 µg mL^−1^. (g) Raman spectra of MoS_2_ and Pt–MoS_2_.

The synthesis process of Pt–MoS_2_ composites is shown in Fig. 1a. Firstly, MoS_2_ nanoflakes were obtained from MoS_2_ powder using the LPE method in an ice-water bath. Then, Pt–MoS_2_ was formed by *in situ* reduction of platinum salts on the obtained MoS_2_ nanoflakes at 60 °C. To improve the stability of nanocomposite, a non-toxic, colorless, water-soluble and non-ionic polymeric compound, which has been widely used as a stabilizer for noble metal nanomaterials with a wide range of sources and low cost, was added as a stabilizer during the synthesis process.^34–36^ In this process, defects and edges on the surface of MoS_2_ containing free sulfur are the main crystalline species for Pt nuclei, and subsequently the Pt nanoparticles can be formed spontaneously on MoS_2_ by redox reaction between MoS_2_ and PtCl_6_^2−^. After preparation, the prepared MoS_2_ and Pt–MoS_2_ were observed using transmission electron microscopy (TEM). As shown in Fig. 1b, the prepared MoS_2_ exhibits a 2D nanosheet structure with uniform dispersion. For Pt–MoS_2_, the TEM image shows that Pt nanoparticles has been well dispersed on MoS_2_ with high density (Fig. 1c). Meanwhile, Pt tends to exist in the form of nanoclusters with relatively irregular shapes (Fig. 1d). Dynamic light scattering (DLS) results in Fig. 1e showed that the size of Pt–MoS_2_ (255.1 ± 29.8 nm) was slightly larger than that of MoS_2_ nanosheets (220.9 ± 21.4 nm) due to the doping of Pt. The UV-vis absorption spectra of MoS_2_ and Pt–MoS_2_ were further investigated (Fig. 1f). The UV-vis absorption spectra of MoS_2_ has two characteristic absorption peaks located at 610 and 670 nm, which are characteristic of layered 2H–MoS_2_.^37^ After Pt decoration, the UV-vis absorption of Pt–MoS_2_ is significantly enhanced in the near-infrared region around 808 nm, which will contribute to the photothermal conversion capability. The Raman spectra of MoS_2_ and Pt–MoS_2_ are illustrated in Fig. 1g. Two characteristic peaks attributed to the E_2g_ and A_1g_ modes of MoS_2_ are observed at 380 cm^−1^ and 407 cm^−1^ for MoS_2_. In addition, these peaks were left-shifted by about 5 cm^−1^ after Pt decorated pristine MoS_2_, indicating the presence of Pt doping.^31^

**Fig. 2:**
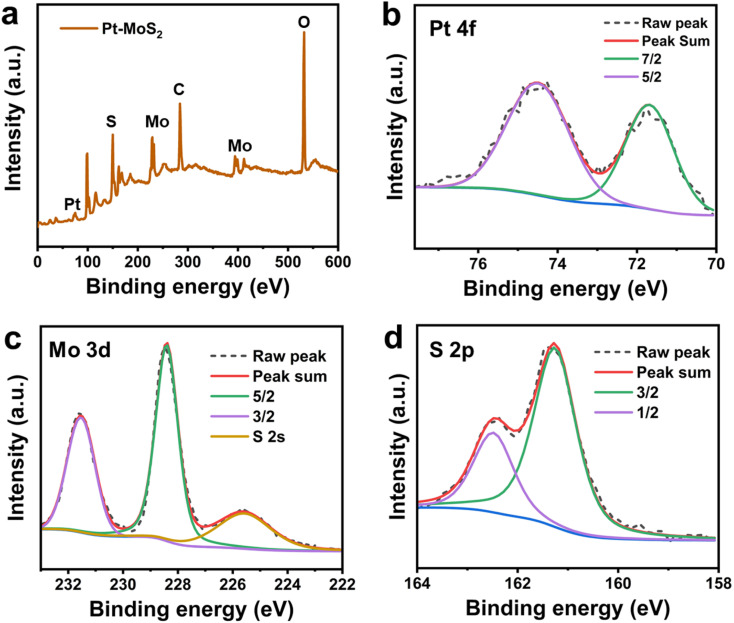
(a) The XPS survey of Pt–MoS_2_. The high-resolution XPS spectra of Pt 4f (b) Mo 3d (c) and (d) S 2p signals for Pt–MoS_2_.

The chemically elemental properties of the synthesized hybrid materials were further investigated by X-ray photoelectron spectroscopy (XPS). The XPS survey of Pt–MoS_2_ shows the presence of Mo, S, and Pt elements, which further proves the successful preparation of the composites (Fig. 2a). Fig. 2b–d shows the high-resolution XPS spectra of Pt, Mo, S peaks of the Pt–MoS_2_ material. The results show that the Pt 4f peaks assigned to Pt 4f_7/2_ (71.88 eV) and Pt 4f_5/2_ (74.58 eV) confirm the presence of zero-valent metallic Pt nanoparticles in Pt–MoS_2_.^38^ In addition, the binding energies of the Mo peaks are located at 228.59 and 231.72 eV are attributed to the Mo 3d_5/2_ and Mo 3d_3/2_ orbitals of Mo^4+^. and The S 2p peaks located at 162.41 and 161.27 eV are attributed to the S 2p_1/2_ and S 2p_3/2_ orbitals of divalent S^2−^.

### 3.2. NIR photothermal performance of Pt–MoS_2_

**Fig. 3:**
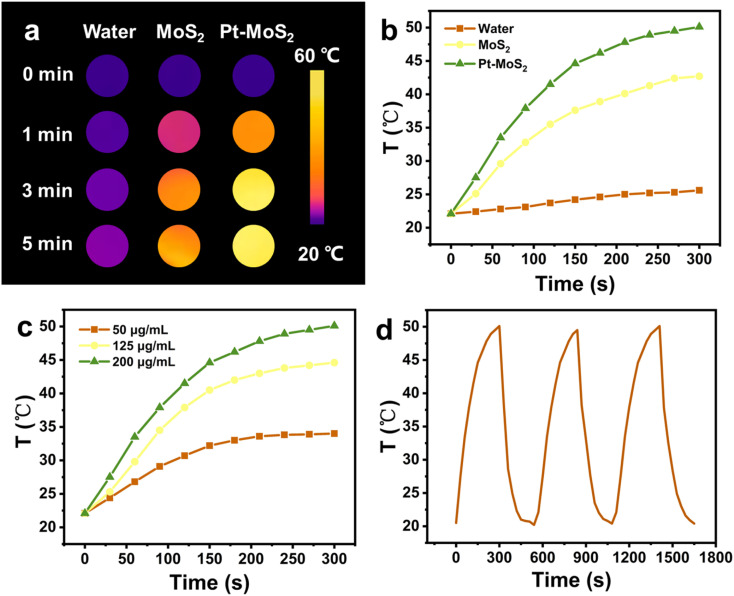
Thermal images (a) and temperature profiles (b) of water, MoS_2_ and Pt–MoS_2_ at same concentration of 200 µg mL^−1^ in 300 s under 808 nm NIR light (1 W cm^−2^). (c) Temperature profiles Pt–MoS_2_ at different concentrations in 300 s under 808 nm NIR light (1 W cm^−2^). (d) Temperature profiles Pt–MoS_2_ at concentration of 200 µg mL^−1^ for 3 cycles under 808 nm NIR light (1 W cm^−2^).

As previously reported, 2D MoS_2_ has good NIR photothermal conversion capability to absorb NIR laser and convert it into heat in a concentration dependent manner.^39^ In addition, Pt has also been shown to be a good photothermal material, and thus the Pt–MoS_2_ composite is expected to exhibit enhanced photothermal conversion capability. Here, we first explored the photothermal properties of the two materials after NIR laser (808 nm) irradiation. As shown in Fig. 3a and b, the maximum temperature difference between Pt–MoS_2_ and MoS_2_ obtained after 5 min of irradiation at the same concentration was 9.7 °C, whereas the temperature rise of water was only 3.8 °C, suggesting that Pt–MoS_2_ possesses a significantly enhanced photothermal conversion capability in the NIR region. In addition, as the concentration of Pt–MoS_2_ increases, the corresponding solution temperature gradually increases, showing a concentration-dependent photothermal effect (Fig. 3c). Furthermore, the Pt–MoS_2_ composites showed good NIR photothermal stability after three “heating–cooling” cycles, indicating their great potential for photothermal therapy (Fig. 3d).

### 3.3. Nanozyme activity of Pt–MoS_2_

**Fig. 4:**
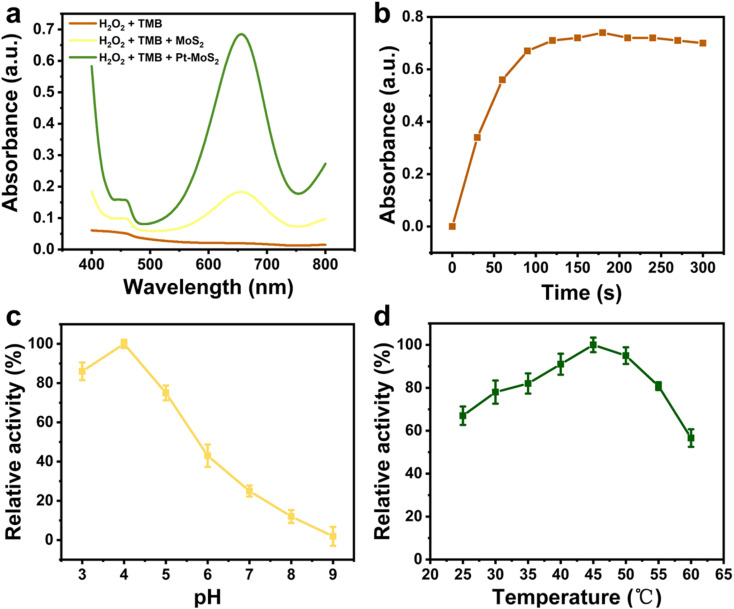
(a) UV-vis absorption spectra of different H_2_O_2_ + TMB solutions containing blank, MoS_2_ or Pt–MoS_2_ after reaction for 10 min. (b) Time-dependent profiles of Pt–MoS_2_ + H_2_O_2_ + TMB solutions at the absorbance of 652 nm in 300 s. Effect of pH (c) and temperature (d) on the POD-mimic catalytic performance.

Subsequently, the POD mimetic ability of the nanomaterials was further investigated by UV spectroscopy through catalysis of chromogenic oxidation of 3,3′,5,5′-tetramethylbenzidine (TMB) with the assistance of H_2_O_2_. Nanozymes with POD-like activity can efficiently catalyze the decomposition of H_2_O_2_ and produce hydroxyl radicals to catalyze the oxidative discoloration of TMB, and thus the occurrence of the oxidation peak of TMB at 652 nm could be an indicator to judge the POD-like catalytic ability. As shown in Fig. 4a, both MoS_2_ and Pt–MoS_2_ can catalyze the TMB oxidation with the assistance of H_2_O_2_, and the POD-like ability of Pt–MoS_2_ is more effective. In contrast, TMB with H_2_O_2_ has almost no absorption at 652 nm in the absence of materials. Moreover, the Pt–MoS_2_-catalysed reaction could reach the reaction equilibrium after 100 s, indicating the efficient catalytic ability of the composite material (Fig. 4b).

Similar to natural enzymes, the POD-like activity of nanozyme is also pH-/temperature-dependent.^40^ As shown in Fig. 4c, the POD-like activity of Pt–MoS_2_ increased with the pH value increase from 3.0 to 4.0, and then gradually decreased with the pH value increase from 4.0 to 9.0, while the relative POD-like activity remained above 50% in the near-neutral pH range. As shown in Fig. 4d, Pt–MoS_2_ has a good thermal stability with an optimum temperature of 45–50 °C. Therefore, the effect of the catalytic system can be amplified to the ideal range by combining the temperature increase due to the photothermal effect.

**Fig. 5:**
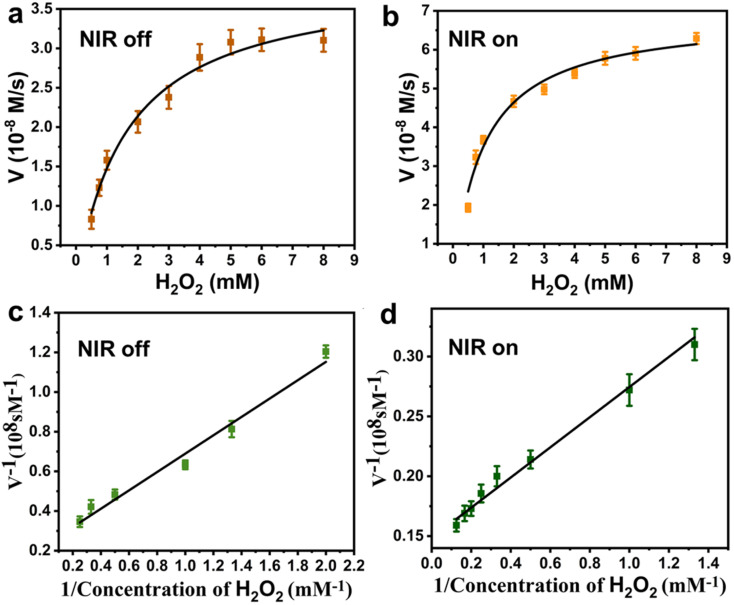
Steady-state kinetic plots of Pt–MoS_2_ using H_2_O_2_ as substrate in the absence (a) and presence (b) of 808 nm NIR light irradiation (1W cm^−2^, 5 min). Double-reciprocal plots of Pt–MoS_2_ using H_2_O_2_ as substrate in the absence (c) and presence (d) of 808 nm NIR light irradiation (1 W cm^−2^, 5 min).

Further, the NIR-enhanced POD-like catalytic mechanism of the Pt–MoS_2_ composites was verified by steady-state kinetic approach. As shown in Fig. 5a and b, there was a clear correlation between the initial reaction velocity (*V*) and H_2_O_2_ substrate concentration whether applying NIR laser irradiation or not. The kinetic parameters of the nanozyme were evaluated using Lineweaver–Burk plot method. As shown in Fig. 5c and d, the reciprocal of substrate concentration showed a direct linear relationship with the reciprocal of *V*. By the classical Lineweaver–Burk equation, the Michaelis–Menten constant (*K*_m_) and the maximum reaction velocity (*V*_max_) were calculated to be 2.046 mM and 4.42 × 10^−8^ M S^−1^, respectively. At the same time, we also tested *K*_m_ and *V*_max_ values using TMB, another substrate for POD-like nanozyme (Fig. S2†) The *K*_m_ and *V*_max_ values of Pt–MoS_2_ for TMB substrate were calculated to be 0.157 mM and 8.49 × 10^−8^ M S^−1^. Generally, lower *K*_m_ values means higher affinity between substrate and POD-like nanozyme, while higher *V*_max_ values mean a better catalytic ability.^17^ As shown in Table 1, Pt–MoS_2_ has good substrate affinities for both H_2_O_2_ and TMB superior to MoS_2_, which is attributed to the composited nanostructure with synergistic active catalytic sites. Although there are several MoS_2_-based composite nanozyme own lower *K*_m_ value, the Pt–MoS_2_ shows the advantages of facile preparation without high temperature, high pressure and organic solvent synthesis conditions. Besides, we first demonstrated that the catalytic parameters of Pt–MoS_2_ nanozyme can be further regulated after NIR laser irradiation. The NIR laser could improve the substrates affinity and catalytic ability. The above results indicate that Pt–MoS_2_ has a synergistic mechanism of photothermal effect and enhanced POD mimetic activity modulated by NIR light, which is expected to be widely used in biomedical fields.

**Table 1:** Comparison of *K*_m_ and *V*_max_ value of POD enzymes with the previous work

Enzyme	*K*_m_, H_2_O_2_ (mM)	*V*_max_ × 10^−8^ (M S^−1^)	*K*_m_, TMB (mM)	*V*_max_ × 10^−8^ (M S^−1^)	Ref.
Pt–MoS_2_	2.046	4.42	0.157	8.49	This work
Pt–MoS_2_ + NIR	0.846	6.73	0.109	12.35	
HRP	3.70	8.71	0.434	10	41
Fe_3_O_4_	154	9.78	0.098	3.44	41
MoS_2_	2.812	8.01	0.357	38.8	42
Fe@MoS_2_	0.03	2.01	—	—	43
N-doped MoS_2_	0.4459	4.348	0.7916	1.796	44
Fe_3_O_4_@MoS_2_–1% Ag	1	18.2	0.19	11.2	45
UiO-66-NH–CO–MoS_2_	0.23	15.7	2.35	0.157	27

### 3.4. Antibacterial activity evaluation of Pt–MoS_2_

**Fig. 6:**
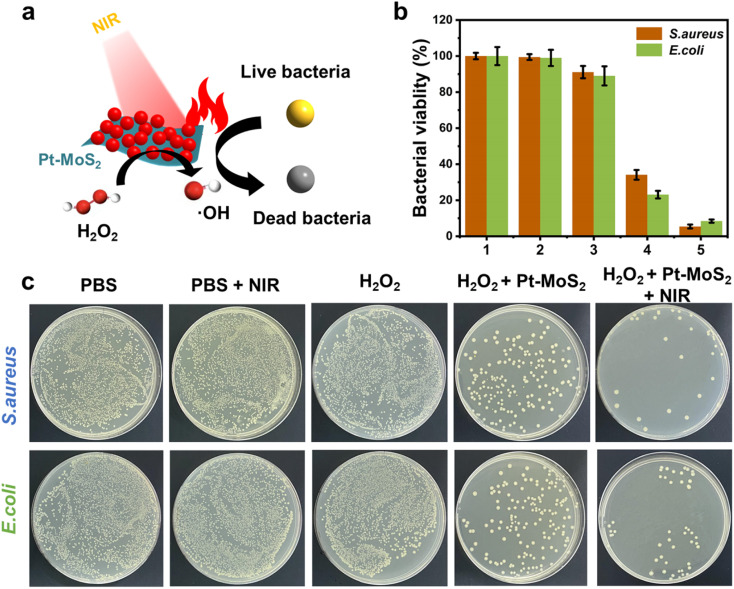
(a) Scheme for the bactericidal mechanism of Pt–MoS_2_ nanozyme after the irradiation of 808 nm NIR laser. (b) Bacterial viabilities of *S. aureus* and *E. coli* after treated by different groups (1: PBS; 2: PBS + NIR; 3: H_2_O_2_; 4: H_2_O_2_ + Pt–MoS_2_; 5: H_2_O_2_ + Pt–MoS_2_ + NIR). (c) Bacterial colonies photographs of *S. aureus* and *E. coli* after treated by different groups.

Inspired by the highly effective photothermal effect, POD-like activity, and NIR-enhanced enzyme-like activity of Pt–MoS_2_ nanocomposites, we hypothesized that Pt–MoS_2_-mediated photothermal-catalytic synergistic therapy could serve as a novel antibacterial strategy for disinfection. Specifically, stimulated by 808 nm near-infrared light, Pt–MoS_2_ nanomaterials can exhibit enhanced POD-like activities that efficiently catalyze the H_2_O_2_ decomposition and form more toxic ˙OH for antibacterial use, and further amplify the therapeutic effect in combination with photothermal therapy (Fig. 6a). Here, to evaluate the antibacterial effect, two methods including optical density and plate counting was used to test the bacterial viability by common *S. aureus* and *E. coli* as Gram-positive and negative bacteria model, respectively. As revealed in Fig. 6b and c, the PBS group alone or the NIR laser group did not produce an inhibitory effect on bacterial growth. H_2_O_2_, which is widely used in the clinic, has a certain effect of sterilization and disinfection. For comparison, Pt–MoS_2_ nanozyme exhibits a certain degree of antibacterial properties due to its intrinsic POD-like activity, which can further catalyze the decomposition of H_2_O_2_ to produce more toxic ˙OH to kill bacteria. Notably, the Pt–MoS_2_ material with enhanced POD-like activity showed 95% antibacterial activity against both *S. aureus* and *E. coli* under the stimulation of 808 nm NIR light. The above results indicate that the novel therapy based on Pt–MoS_2_ have broad-spectrum antibacterial properties. By further increasing the dose of Pt–MoS_2_ or the laser power, the bacteria are expected to be completely inhibited. Due to the wide application of photothermal therapy in clinical practice and the convenience of laser equipment, we believe that this new combination therapy has broad application prospects in the efficient sterilization and treatment of other diseases.^46^

**Fig. 7:**
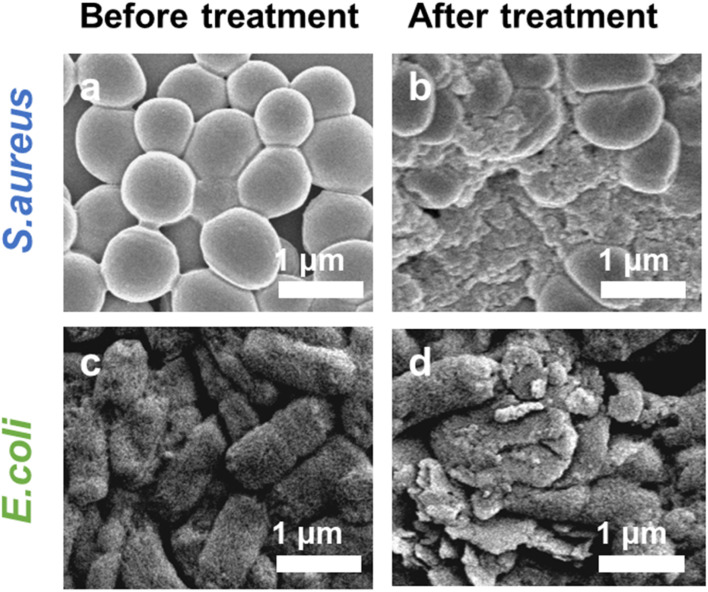
The representative SEM images of *S. aureus* before (a) and after (b) treatment with H_2_O_2_ + Pt–MoS_2_ + NIR. The representative SEM images of *E. coli* before (c) and after (d) treatment with H_2_O_2_ + Pt–MoS_2_ + NIR.

Further, in order to investigate the bactericidal effect and mechanism of the synergistic therapy, the bacterial morphology before and after various treatments was observed by scanning electron microscope (SEM). As indicated in Fig. 7a and b, *S. aureus* before treatment had smooth surfaces and retained intact morphological features, whereas the outer membranes of the bacteria treated with the combination therapy were damaged and appeared distinctly wrinkled, corroborating the highly effective bactericidal effect of H_2_O_2_ + Pt–MoS_2_ + NIR. Moreover, similar results was obtained for *E. coli* (Fig. 7c and d). Further, we performed SYTO/PI live-dead staining experiments. Live bacteria could be labelled by SYTO green fluorescent dye, while damaged bacteria showed red fluorescence after labelling by PI dye (Fig. S3†). When treated with PBS buffer, most of the bacteria remained alive and therefore had no red fluorescence signal. However, when the bacteria were exposed to the H_2_O_2_ + Pt–MoS_2_ + NIR system, most of the bacteria were destroyed, resulting in a significant enhancement of the red fluorescence signal. The above experimental results suggest that Pt–MoS_2_ nanozyme can be used as an efficient antibacterial agent and further can amplify the efficacy under the combined effect of NIR light.

### 3.5. Biological compatibility evaluation of Pt–MoS_2_

**Fig. 8:**
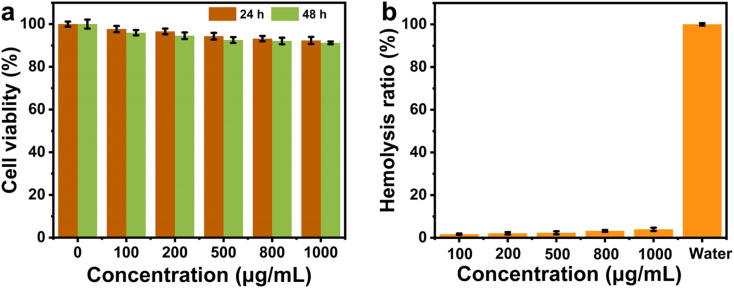
NIH3T3 cell viability (a) and hemolysis rate (b) of Pt–MoS_2_ at different concentrations.

To explore the practical potential of the novel antibacterial materials for bioapplications, biosafety and biocompatibility were further evaluated. By selecting common fibroblasts as a model, the effect of Pt–MoS_2_ on cell survival at different concentrations was tested by MTT method. As demonstrated in Fig. 8a, the nanohybrids had negligible cytotoxicity and excellent cytocompatibility. The hemolysis experiments further showed that even at the concentration of 1000 µg mL^−1^, the Pt–MoS_2_ material exhibited extremely low hemolysis rate (<4%) (Fig. 8b). The above experiments demonstrate the good biocompatibility of Pt–MoS_2_, which is expected to be used for *in vivo* antimicrobial and sterilization treatments in the future.

## 4. Conclusions

In conclusion, Pt–MoS_2_ composites synthesized by self-reduction method with a simple and green approach have been successfully developed as a novel NIR-enhanced POD-like nanozyme. The successful preparation of Pt–MoS_2_ was demonstrated using various characterization techniques. Thanks to the Pt modification, Pt–MoS_2_ composite exhibits significantly enhanced photothermal and POD catalytic activities over bulk MoS_2_, indicating the significant impact of the formation of metal heterostructures in improving the performance of conventional 2D materials. The novel nanozymes can achieve efficient synergistic antibacterial activity by NIR-triggered enhanced POD catalytic therapy and photothermal therapy. The experimental results showed that the synergistic antibacterial material was not only effective in eliminating *S. aureus* infections, but also highly cytocompatible and hemocompatible. Overall, this work provides a new strategy to enhance the activity of nanozymes and develop a rapid and efficient method to inhibit bacterial infections, which is expected to replace antibiotics and prevent the spread of bacterial resistance. We believe the clinical application of photothermal therapy by improving the properties of laser devices and nanomaterials can be further enhanced from the laboratory to the clinic, opening up new possibilities for the treatment of a wide range of diseases.

## Data availability

Data will be available on request.

## Author contributions

Xia Feng conceived the project and supervised the research. Xia Feng propose the idea of methods. Liangyu Li and Lidong Cao designed and performed the experiments. Yueqin Zhang, Yumeng Liu, Yaojun Wang, and Xiao Wang discussed the data and check the manuscript; Liangyu Li and Lidong Cao wrote the paper.

## Conflicts of interest

The authors declare no conflict of interest.

## Supplementary Material

RA-014-D4RA05487C-s001
