# Supplementary material for: Synthesis of Pt–MoS2 with enhanced photothermal and peroxidase-like properties and its antibacterial application
Source: RSC Adv. 2024 Sep 18;14(40):29428–38. doi: 10.1039/d4ra05487c (PMC11409452; doi:10.1039/d4ra05487c)
Supplement: RA-014-D4RA05487C-s001 [file RA-014-D4RA05487C-s001.pdf]

Supplementary Information (SI) for RSC Advances.
This journal is © The Royal Society of Chemistry 2024

Supporting information

Synthesis of Pt-MoS_2 with Enhanced Photothermal and Peroxidase-like Properties and Its Antibacterial Application

Liangyu Li[a], *Yueqin Zhang*[d], *Yumeng Liu*[d], *Yaojuan Wu*[a], *Xiao Wang*[d], *Lidong Cao*[b,c*], *Xia Feng*[a,b*]

a Nursing Department, Zhejiang Provincial People's Hospital, Affiliated People's Hospital, Hangzhou Medical College

b Department of Hepatobiliary & Pancreatic Surgery and Minimally Invasive Surgery, Zhejiang Provincial People's Hospital, Affiliated People's Hospital, Hangzhou Medical College

c College of Mechanical Engineering, Zhejiang University, Hangzhou, China

d School of Public Health, Hangzhou Medical College, Hangzhou, China

* Correspondence: fx19961219@163.com (X.F.); ilkwenxu2019@sina.com (L.C.)

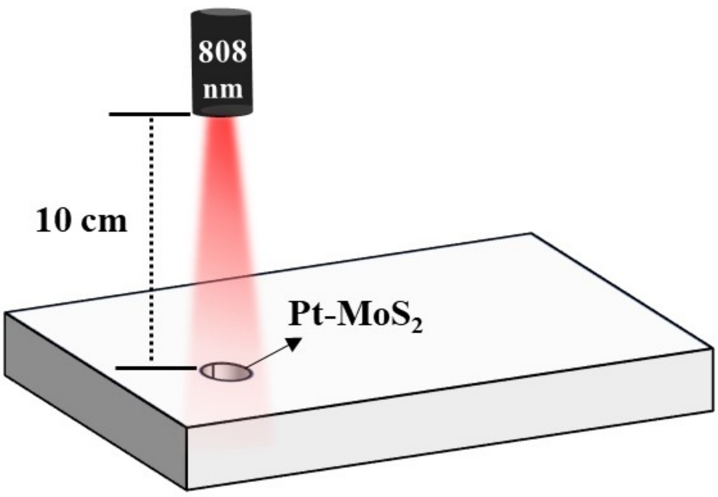

Fig. S1. Schematic illustration for the setup to test photothermal property.

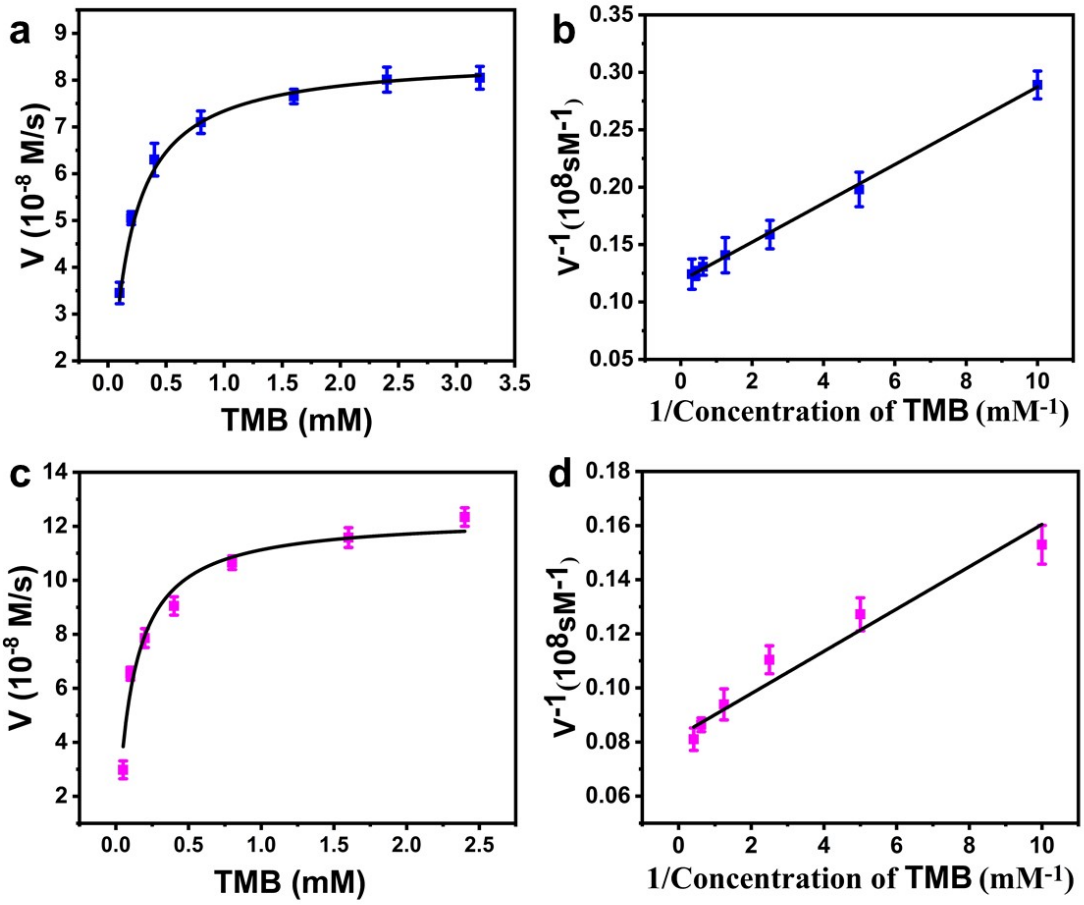

Fig. S2. Steady-state kinetic plots and Double-reciprocal plots of Pt-MoS_2 using TMB as substrate in the absence (a-b) and presence (c-d) of 808 nm NIR light irradiation (1W/cm^2, 5 min).

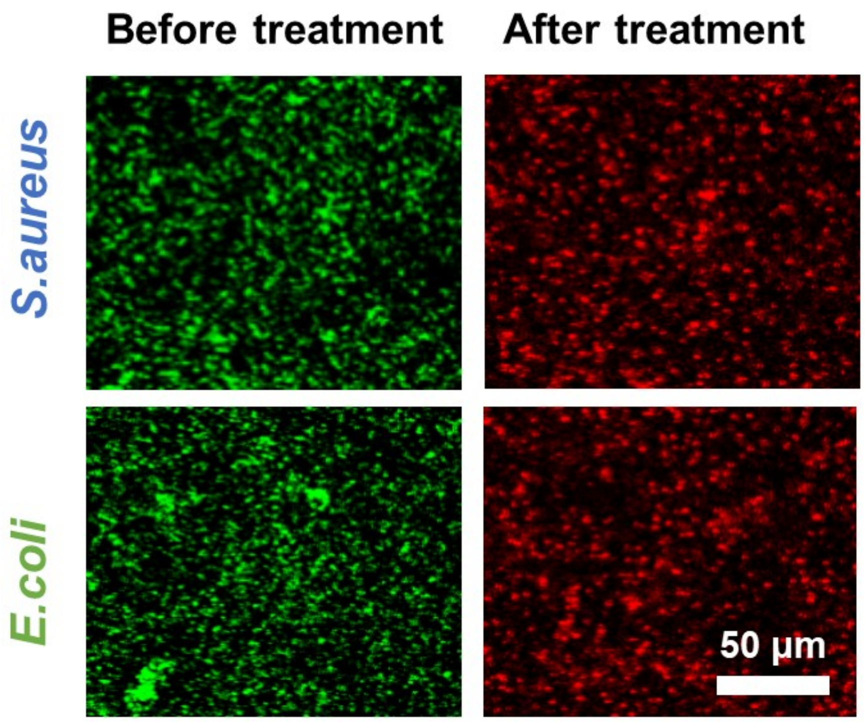

Fig. S3. The representative SYTO/PI live-dead staining fluorescent images of *S.aureus* and *E.coli* before and after treatment with H_2O_2 + Pt-MoS_2 + NIR.
